# A randomized safety study of tolerance to table rotation in dynamic trajectory radiotherapy in healthy volunteers

**DOI:** 10.1016/j.phro.2025.100796

**Published:** 2025-06-14

**Authors:** Paul-Henry Mackeprang, Jenny Bertholet, Claas Wessels, Jean-Benoit Rossel, Andreas Limacher, Daniel M. Aebersold, Michael K. Fix, Peter Manser

**Affiliations:** a Division of Medical Radiation Physics and Department of Radiation Oncology, Inselspital, Bern University Hospital and University of Bern, 3010 Bern, Switzerland; b Varian Medical Systems Imaging Laboratory GmbH, 5405 Baden, Switzerland; c Department of Clinical Research, University of Bern, 3012 Bern, Switzerland

**Keywords:** Dynamic trajectory radiotherapy, DTRT, Non-coplanar radiotherapy, VMAT, Safety study, Motion sickness

## Abstract

This study aimed to show that table movement of dynamic trajectory radiotherapy (DTRT) does not induce more motion sickness than standard-of-care non-coplanar volumetric modulated arc therapy (ncVMAT). Forty-one healthy volunteers underwent dry-runs of DTRT and ncVMAT in four different, randomly allocated sequences. The primary outcome was the Motion Sickness Assessment Questionnaire (MSAQ) summary score. The average change in MSAQ summary score before to after dry-runs was 1.88 for DTRT and 1.62 for ncVMAT. The difference between both techniques was 0.26 [95% CI: −0.24 to 0.75]; the CI demonstrated non-inferiority of DTRT to ncVMAT for motion sickness in healthy volunteers.

## Highlights

• Motion sickness in Dynamic Trajectory Radiotherapy was non-inferior to non-coplanar VMAT.
• Over 164 dry runs of both techniques, only one symptomatic motion sickness occurred.
• Yet, both techniques led to increased motion sickness scores in questionnaires.

## 1 Introduction

The current standard-of-care in radiotherapy is coplanar intensity modulated radiotherapy and volumetric modulated arc therapy (VMAT) with the patient lying on a treatment table that remains static during beam-on. Interest has grown for non-coplanar techniques, which can improve dosimetric plan quality. However, currently, non-coplanar treatments are only delivered with the beam off during table rotation, e.g. non-coplanar VMAT (ncVMAT) [1].

Dynamic trajectory radiotherapy (DTRT) is an investigational technique that extends VMAT with dynamic rotation of the treatment table and collimator during beam-on [2]. DTRT was investigated for multiple treatment sites [[3], [4], [5], [6], [7], [8], [9], [10], [11]]. In particular for head and neck cancer, planning studies showed improved dosimetry with DTRT over VMAT [12,13]. With an appropriate collision model, DTRT plans were deliverable with high mechanical and dosimetric accuracy [2,12,[14], [15], [16]]. Yet, safety for patient treatments has not been investigated. Dynamic movement of the treatment table has been suspected to cause motion sickness in other investigational radiotherapy techniques [[17], [18], [19], [20]].

This study aimed to show that dynamic table rotation of DTRT does not induce more motion sickness than standard-of-care ncVMAT in healthy volunteers.

## 2 Materials and methods

Subjects underwent multiple dry runs of DTRT and ncVMAT in an open-label randomized two-procedure four-sequence four-period crossover trial was conducted in February 2023 over two weeks at Inselspital Bern. The trial was approved by the research ethics committee of Bern (BASEC-ID 2022-02025) and registered in the Swiss national clinical trial portal (SNCTP 000005333). Details of trial oversight and responsibilities are given in Supplementary material A.

Eligible subjects were healthy volunteers. Full inclusion and exclusion criteria are listed in Supplementary Table S1. Enrollment was open to employees of Inselspital Bern and Varian. Between January 1st and March 1st, 2023, 48 volunteers were screened for participation and 41 were enrolled into the study after giving informed consent. The Intention-to-treat (ITT) population included 41 volunteers. Supplementary Fig. S2 shows the study recruitment flow-chart.

Each volunteer underwent fitting of a 3-point thermoplastic mask and underwent four dry runs of a simulated (no radiation applied) radiotherapy treatment for oropharyngeal cancer. Two were DTRT (intervention, D) and two were ncVMAT (control, V), in a replicate crossover design. Volunteers were randomized at time of inclusion in a 1:1:1:1 ratio into one of four groups, with each group following a different sequence of the four runs (DVVD, VDDV, DDVV, VVDD) with a wash-out period of 45 min between runs. To address potential biases influenced by the sequential order of the run, rather than the technique, a replicate crossover design was adopted. Firstly, the majority of the volunteers had no prior experience on a treatment table or the radiotherapy bunker environment. Secondly, volunteers were immobilized by a thermoplastic head mask. Thirdly, the state in which volunteers would arrive for their dry runs was undefined, potentially increasing their susceptibility to motion sickness. Fourthly, any bias from insufficiently long wash-out periods was mitigated. Each dry run lasted 6–10 min including setup time. Volunteers performed all four dry runs on the same day. Supplementary Fig. S3 shows the study flow-chart.

The primary outcome of this study was motion sickness. Each volunteer filled in a Motion Sickness Assessment Questionnaire (MSAQ) before and after each dry run [21]. The MSAQ consists of 16 items assessing symptoms of motion sickness on a scale from 1 (not at all) to 9 (severely). The MSAQ summary score was computed by summing up all 16 items for a total score between 16 and 144. The main endpoint was changes of the MSAQ summary score after to before dry runs. This endpoint was tested for non-inferiority of DTRT dry runs to ncVMAT dry runs. Secondary outcomes included MSAQ sub-scores for gastro-intestinal, central nervous, peripheral nervous and sopite-related symptoms [21] and clinically relevant motion sickness, defined as an MSAQ summary score of ≥80 or any score ≥7 in any single item after a dry run [18].

All dry runs were performed on a TrueBeam system (Varian, Palo Alto, USA). Supplementary Fig. S4 shows the beam configuration of the treatment plans used for all DTRT (A) and all ncVMAT (B) dry runs in a 3D view. The DTRT plan used two trajectories with continuous gantry and table motion, each covering 360° of gantry range. The ncVMAT plan used four half-arcs at −20°, 20° and 70° discrete table angle and a 200° partial arc at −70° table angle. Both plans were based on an oropharyngeal cancer case used in a previous planning study [12]. The maximum angular speed of our TrueBeam was 6 °/s for gantry, 3 °/s for table. During dry runs, the slowest machine component set the pace. In DTRT planning, users can set the maximum gantry-table rotation gradient defined as the ratio of the change in table angle to the change in gantry angle between two control points [22]. In this study, this gradient was set to 0.5 °/°. Log files were recorded every 20 ms and resampled to 100 ms for analysis to reduce jitter. For ncVMAT, the maximum table speed is used to manually rotate the table between arcs with no log files available.

Based on power analysis, the enrollment target was set at 40 volunteers. Statistical analysis was performed using generalized linear mixed models with each respective endpoint as outcome. Details are given in supplementary material B and C. All analyses were done in Stata version 18.0 [23] and R version 4.2.2 (October 31, 2022) [24].

## 3 Results

Study demographics are shown in Supplementary Table S5. The study population showed a 3:2 male predilection, mirroring typical oropharyngeal cancer patient populations. Age differed from similar patient cohorts (median of 40 years). Median height of the volunteers was 175 cm (lower and upper quartiles 164 cm and 181 cm) and median weight was 69 kg (lower and upper quartiles of 60 kg and 85 kg). Thirteen volunteers reported a history of motion sickness.

**Fig. 1:**
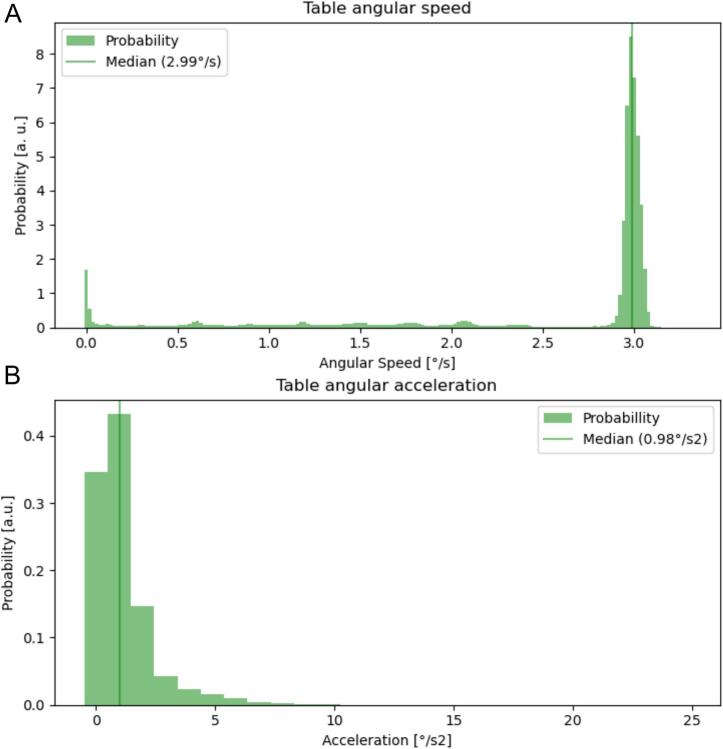
A: Angular Speed and acceleration of the table over all DTRT dry runs as extracted from trajectory log files. The median table angular speed is close to its maximum of 3°/s. B: During DTRT deliveries, the median angular acceleration was 1.0°/s^2^ with higher values reached at the start and end of each trajectory.

The median angular speed of the table in DTRT dry runs was 3.0 °/s (lower and upper quartiles 2.9 °/s and 3.0 °/s). Median table acceleration was 1.0 °/s^2^ (lower and upper quartiles 0.0 °/s^2^ and 1.0 °/s^2^. Fig. 1 shows histograms of both angular speed and acceleration.

The mean change in MSAQ summary score was 1.88 [95 % CI 0.79 to 2.97] for DTRT and 1.62 [0.78 to 2.46] for ncVMAT in the ITT population. The difference between techniques was 0.26 [-0.24 to 0.75]. Table 1 summarizes average changes and differences in MSAQ summary score, results in the per-protocol population are given in Supplementary Table S6. The confidence intervals for the difference, on both sets, were below the non-inferiority margin of 4 points. We hence concluded that DTRT is non-inferior to ncVMAT in terms of MSAQ summary scores. Both DTRT and ncVMAT led to a significant increase in both central and sopite-related sub-scores, but a slight but significant decrease in the peripheral sub-score (Table 1). Summary measures of the MSAQ score and model coefficients are reported in Supplementary Figs. S7–S9 and Tables S10–S12.

**Table 1:** Analysis of the MSAQ summary score and sub-scores on the intention-to-treat (ITT) population (n = 41). We show the average change for the summary and each sub-score with both techniques, and the difference between them. 95 % confidence intervals are based on robust standard errors. MSAQ: Motion sickness assessment questionnaire. DTRT: Dynamic trajectory radiotherapy. ncVMAT: Non-coplanar volumetric modulated arc therapy.

Average [95 % CI] change: MSAQ summary score (before to after)			

	**DTRT**	**ncVMAT**	**Difference**

Summary score	1.88 [0.79 to 2.97]	1.62 [0.78 to 2.46]	0.26 [−0.24 to 0.75]

Average [95 % CI] change: MSAQ sub-scores (before to after)			

Sub-score	**DTRT**	**ncVMAT**	**Difference**

Gastrointestinal	0.21 [−0.02 to 0.45]	0.12 [−0.04 to 0.27]	0.10 [−0.04 to 0.24]
Central	1.30 [0.55 to 2.05]	1.16 [0.58 to 1.74]	0.14 [−0.16 to 0.44]
Peripheral	−0.10 [−0.15 to −0.05]	−0.06 [−0.13 to 0.00]	−0.04 [−0.11 to 0.03]
Sopite-related	0.51 [0.16 to 0.85]	0.38 [0.12 to 0.65]	0.12 [−0.13 to 0.38]

There was one occasion of clinically relevant motion sickness, rating item number 14 (“I felt like I was spinning”) with a score of 7 after the first DTRT dry run, corresponding to 0.6 % (1/164) of all runs and 2.4 % (1/41) of all volunteers. No summary score of 80 or more occurred. One adverse event (0.6 %) occurred with a volunteer reporting vertigo after the second dry run (DTRT). Symptoms were self-limiting and resolved within five minutes after run completion. No adverse events were reported after ncVMAT dry runs. No serious adverse events were reported.

## 4 Discussion

In this randomized crossover trial, motion sickness induction associated with DTRT was non-inferior compared to ncVMAT in healthy volunteers. While both techniques were associated with a statistically significant increase in MSAQ summary scores as well as central and sopite-related sub-scores, overall rates of clinically relevant or symptomatic motion sickness remained low.

Forty-one volunteers completed the study, with one protocol deviation in one volunteer due to technical reasons (machine interlock). Given the experimental nature of DTRT, this protocol completion rate supports the deliverability of DTRT previously shown in phantom measurements [2,11,12,25] and confirms that both DTRT and ncVMAT are well tolerated. Only one occurrence of an elevated item score (“I felt like I was spinning”) and one case of clinically symptomatic motion sickness (vertigo) occurred after DTRT dry runs. Neither required any dry run interruption or medical intervention.

Both techniques showed an increase in overall MSAQ scores, indicating a potential concern for motion sickness in non-coplanar treatment techniques. Similar studies exist for couch tracking [17,18]: Jöhl et al. reported MSAQ scores for 100 volunteers after three one-minute segments of the table compensating for respiratory motion, alternating with static segments and a trailing segment of sinusoid motion. While mean reported scores were low, sum scores were higher than in the current study due to an increased rate of outliers and higher sopite-related scores [2]. D’Souza et al. recruited patients undergoing radiotherapy for various causes, and subjected them to a fixed motion trajectory similar to breathing motion compensation. Overall MSAQ scores were lower than in the current study with a maximum item score of 5, and 95 % of the item scores being 1. Subjects were patients under radiotherapy at the time of study inclusion. Repeated exposure to, or familiarization with, an environment potentially causing motion sickness has been shown to decrease susceptibility to it [26]. Some of the volunteers included in the current study had never been on a treatment table before, a possible reason for the higher scores seen in the current work. In addition, volunteers wore a thermoplastic mask, which may trigger claustrophobia and impair subjects’ vision, potentially increasing motion sickness scores [27]. Buckley et al. and Beyer et al. examined volunteer tolerance to being rotated around their superior-inferior axis inside an MRI scanner, simulating fixed-gantry-rotating-patient radiotherapy delivery [19,20]. Both studies used a fast motion survey [28] and a short form of the state-trait anxiety inventory [29] with the latter adding a modified MSAQ. While in Buckley et al. participants did report significantly increased fast motion survey scores, motion sickness seemed not to be the main cause of discomfort, as in both studies volunteers had to interrupt their runs for discomfort from immobilization.

The cohort of volunteers included into the current study limited the study in several important aspects. Subjects were healthy volunteers not experiencing the (co-)morbidities or psychological implications affecting cancer patients. For logistical reasons, recruitment was limited to employees of Inselspital Bern or Varian, subjecting them to possible conflicts of interest. Employees involved in any way with the project were excluded from participation, with volunteers recruited from a plethora of departments (both medical and non-medical). This also caused the median age to be 20 years lower than that of oropharyngeal cancer patients [30]. The treatment room door was open and personnel present to enable machine motion, which may affect volunteers’ experience. Lastly, as no radiation is applied in dry runs, the slowest mechanical axis sets the pace, not the dose rate. The gantry speed is user-configurable and may vary between systems, but this study uses the highest configurable speed. Because the maximum angular speed of the table is half of that of the gantry, dynamic table tends to slow down dry run and delivery for DTRT compared to ncVMAT.

Despite the inherent limitations of this study, it presents the first data on DTRT with human subjects. Dynamic table rotation during beam-on should continue on its road to clinical implementation, now that its algorithmic pitfalls can be handled in the form of DTRT, which can be delivered safely, accurately, efficiently and with a potential benefit for patients on conventional linacs [2,13,14,16,31].

In conclusion, the study successfully demonstrated that DTRT is non-inferior to ncVMAT in terms of motion sickness in a cohort of healthy volunteers undergoing dry runs of treatments for oropharyngeal cancer. While both techniques are associated with a statistically significant increase in MSAQ scores, overall rates of clinically relevant motion sickness are low (0.6 %). These findings support the safety and tolerability of DTRT in future applications.

## CRediT authorship contribution statement

**Paul-Henry Mackeprang:** Conceptualization, Methodology, Software, Formal analysis, Investigation, Data curation, Writing – original draft, Writing – review & editing, Visualization, Project administration. **Jenny Bertholet:** Conceptualization, Methodology, Validation, Investigation, Data curation, Writing – original draft, Writing – review & editing. **Claas Wessels:** Methodology, Resources. **Jean-Benoit Rossel:** Methodology, Formal analysis, Data curation, Writing – review & editing, Visualization. **Andreas Limacher:** Methodology, Formal analysis, Data curation, Writing – review & editing. **Daniel M. Aebersold:** Conceptualization, Resources, Supervision, Funding acquisition. **Michael K. Fix:** Conceptualization, Methodology, Resources, Writing – review & editing, Supervision, Funding acquisition. **Peter Manser:** Conceptualization, Methodology, Resources, Writing – review & editing, Supervision, Project administration, Funding acquisition.

## Declaration of competing interest

The authors declare the following financial interests/personal relationships which may be considered as potential competing interests: This work was supported by Varian, a Siemens Healthineers Company.

JB, MF, PM declare funding from Grant 200021_185366 of the Swiss National Science Foundation outside of the submitted work.

JBR, AL are affiliated with the Clinical trials unit of the Department of Clinical Research, University of Bern (CTU Bern), which has a staff policy of not accepting honoraria or consultancy fees. However, CTU Bern is involved in design, conduct, or analysis of clinical studies funded by not-for-profit and for-profit organizations. In particular, pharmaceutical and medical device companies provide direct funding to some of these studies. For an up-to-date list of CTU Bern’s conflicts of interest, see https://dcr.unibe.ch/services/declaration_of_interest/index_eng.html.

## Data Availability

Research data are stored in an institutional repository and will be shared upon request to the corresponding author.
