# Supplementary material for: A randomized safety study of tolerance to table rotation in dynamic trajectory radiotherapy in healthy volunteers
Source: Phys Imaging Radiat Oncol. 2025 Jun 14;35:100796. doi: 10.1016/j.phro.2025.100796 (PMC12226396; doi:10.1016/j.phro.2025.100796)
Supplement: Supplementary Data 1 [file mmc1.pdf]

A randomized safety study of tolerance to table rotation in dynamic trajectory radiotherapy in healthy volunteers

Short title: DTRT volunteer study

Paul-Henry Mackeprang[a], Jenny Bertholet[a], Claas Wessels[b], Jean-Benoit Rossel[c], Andreas Limacher[c], Daniel M Aebersold[a], Michael K Fix[a], Peter Manser[a]

[a]Division of Medical Radiation Physics and Department of Radiation Oncology, Inselspital, Bern University Hospital and University of Bern, 3010 Bern, Switzerland
[b]Varian Medical Systems Imaging Laboratory GmbH, 5405 Baden, Switzerland
[c]CTU Bern, University of Bern, 3012 Bern, Switzerland

Supplementary Material

Supplementary material A: Ethical statement, trial oversight, and responsibilities of the different parties

The trial was approved by the research ethics committee of Bern (BASEC-ID 2022-02025) and registered in the Swiss national clinical trial portal (SNCTP 000005333). All volunteers provided written informed consent. Volunteer data and trial conduct were monitored by an independent agency. Trial design, statistical analysis plan, data collection, analysis and interpretation as well as drafting of the manuscript were under the responsibility of Inselspital Bern and the University of Bern, independently of Varian. Varian provided machine time and technical assistance.

Supplementary Table S1

Inclusion Criteria	Exclusion Criteria
• Over 18 years of age<br>• Able to fill-in the required questionnaires in either English or German language.<br>• Body height and weight comply with the mechanical limits of the treatment table (220 cm, 160 kg) of the TrueBeam radiotherapy delivery system (Varian, Palo Alto, CA, USA).<br>• Able to lie down for the required amount of time<br>• Tolerate fixation by thermoplastic mask (as routinely done in head-and-neck radiotherapy treatments).	• Pre-existing conditions that might impair balance or cause vertigo<br>• Pregnancy (pregnancy tests were required for volunteers of child-bearing potential).

Supplementary Figure S2

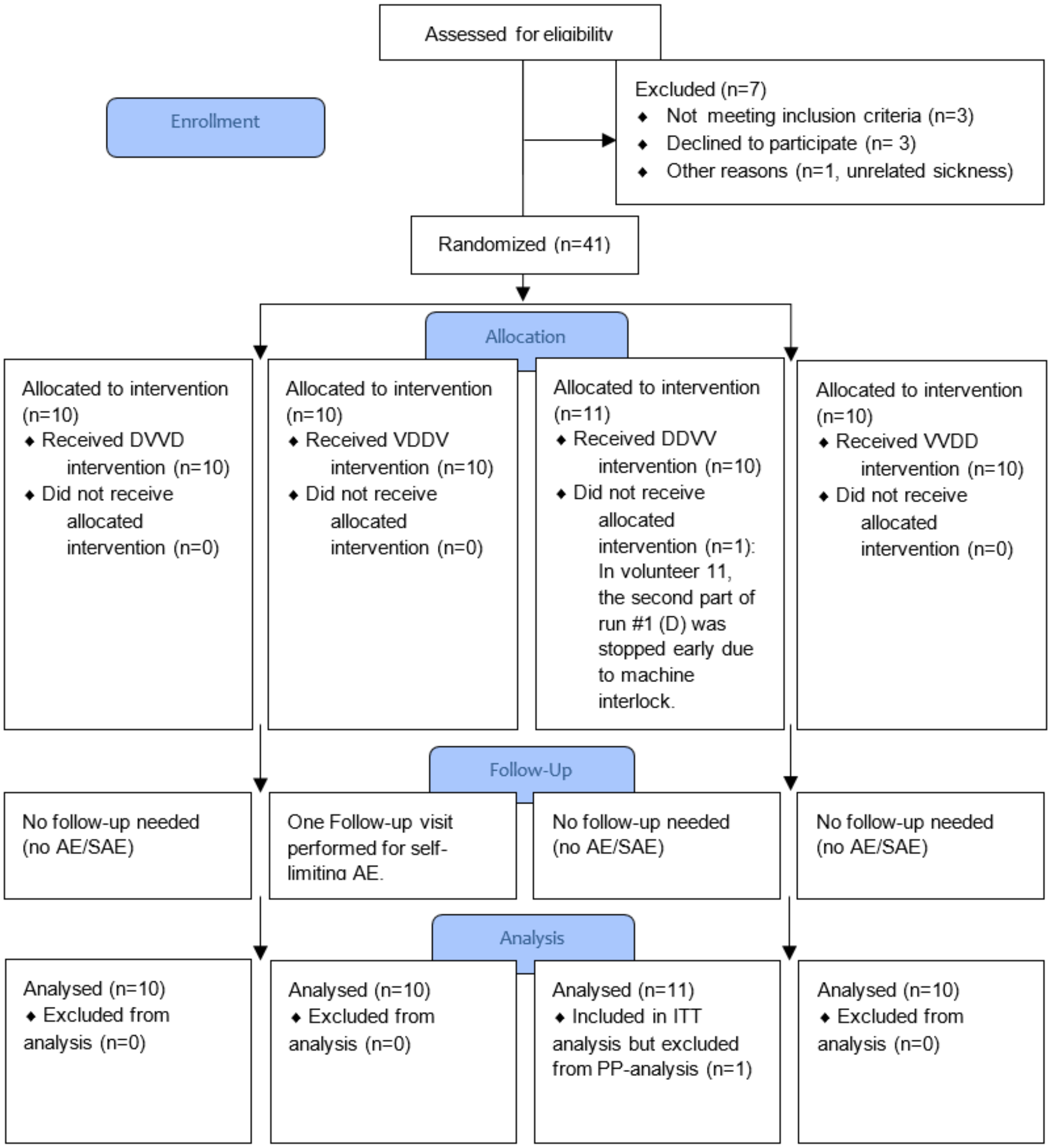

Supplementary Figure S1: Study recruitment flow-chart. One out of forty volunteers had a protocol deviation in the second out of four runs, so one additional volunteer was recruited and randomized into the DDVV arm.

Supplementary Figure S3

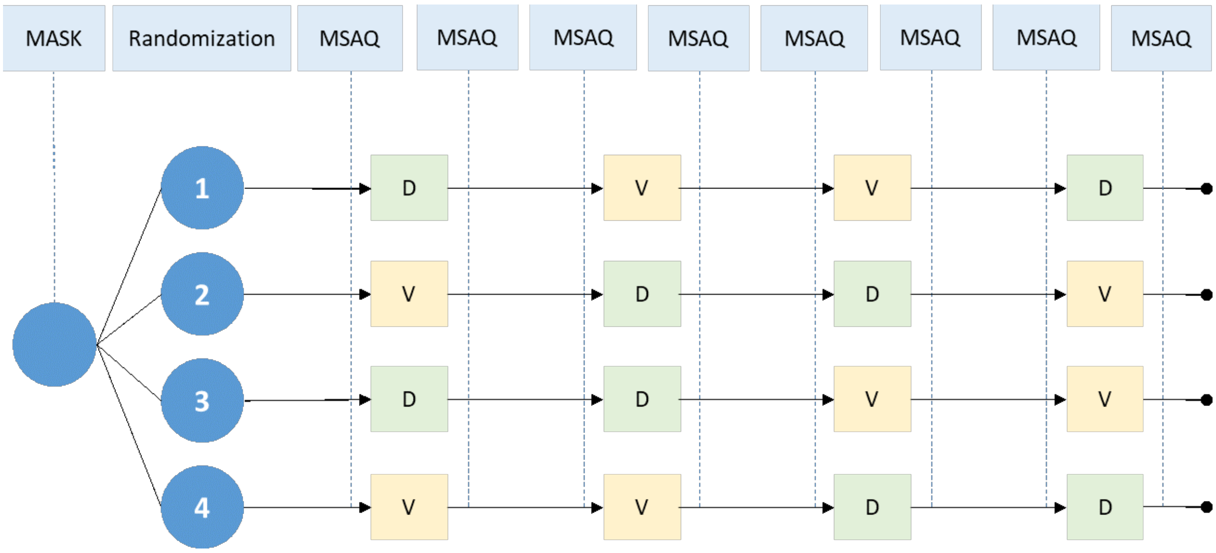

Supplementary Figure S4: Flow-chart of the study design and procedures. 1-4: randomization groups of dry run sequences.
MSAQ: motion sickness assessment questionnaire. D: dry run of dynamic trajectory radiotherapy. V: dry run of non-coplanar volumetric modulated arc therapy.

Supplementary Figure S4

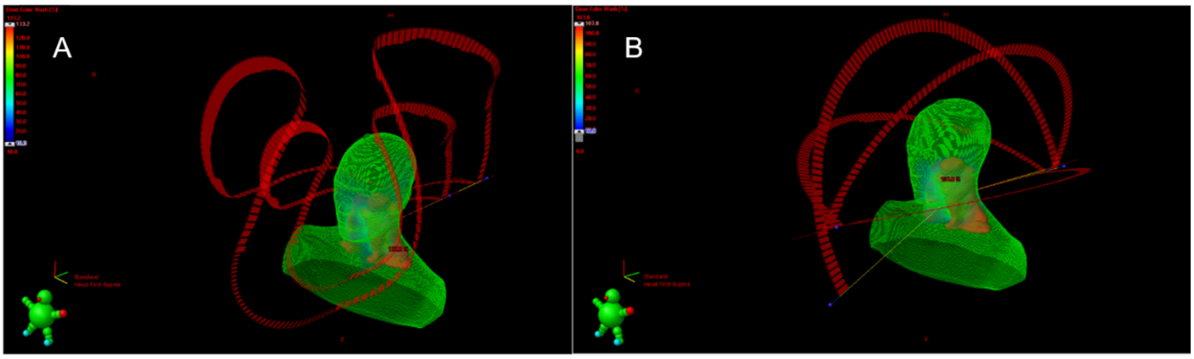

Supplementary Figure S5: 3D-view of treatment plans used for dry runs. Trajectories shown in dashed red for DTRT (A) and ncVMAT (B). Body contour shown in green, planning target volume (PTV), derived from an oropharyngeal cancer case, shown in red inside the body contour.

Supplementary material B: Power analysis

No minimal clinically important difference (MCID) in the MSAQ summary score has been reported in literature. Therefore, we based the non-inferiority margin on clinical reasoning. We regard a difference on the MSAQ summary score of 5 points as clinically relevant, below 5 points as clinically irrelevant. We thus set the non-inferiority margin at 4 points, which corresponds to an effect size of 0.5 based on the standard deviation of 7.6 reported in similar published work [1]. Using a Wilcoxon signed rank test for paired data with a non-normal distribution, we calculated that 34 volunteers were required in total to establish non-inferiority with a power of 80% at a significance level of 0.025 (one-sided). This calculation neglects the

repeat measurement of each technique within a sequence, i.e., the calculation is conservative and the actual power >80%. Dropouts were to be replaced, and randomization groups needed to be balanced. We therefore set the enrollment target at 40 volunteers.

Supplementary material C: Statistical analysis

The intention-to-treat (ITT) population included all volunteers who had undergone randomization and completed at least one dry run. In the per-protocol (PP) analysis, only volunteers who completed all four dry runs without any protocol deviations were evaluated.

For the primary outcome, change in the MSAQ summary score, a generalized linear mixed model with a Gamma distribution and its canonical logarithmic link function and robust standard errors was used with the MSAQ summary score after the dry run as outcome and the following predictors [2]:

1) the MSAQ summary score before the run,
2) an indicator for the technique (DTRT vs. ncVMAT),
3) an indicator for the randomization sequence (DVVD, VDDV, DDVV or VVDD),
4) the number of the dry run (1,2,3, or 4) as a continuous variable.

Repeated measurements for each volunteer, and consequently clustered data, were handled via a random effect at the volunteer level.
Because scores and differences before to after dry runs were asymmetrically distributed (right-skewed) and shifted, we shifted all MSAQ summary scores by -15 and chose a Gamma distribution for model fitting. From this model, we calculated marginal mean changes as well as the marginal effect, i.e. the difference between the two techniques with corresponding 95% confidence intervals. Non-inferiority was considered established if the confidence interval was completely below the non-inferiority margin of 4 points in MSAQ summary score in both the ITT and PP volunteer sets. We assessed normality of model residuals by a QQ-plot, which was satisfactory.

Secondary continuous outcomes (MSAQ sub-scores) were evaluated using the same modelling approach as described above. Both ITT and PP analyses were performed for secondary outcomes. No subgroup analyses or interim analyses were performed. All analyses were done in Stata version 18.0 [3] and R version 4.2.2 (October 31, 2022) [4].

Supplementary Table S5

Supplementary Table S8 shows the study demographics.
AE: Adverse event. SAE: Serous adverse event.

	Total (n = 41)
**Completed all fours runs**	41 (100%)
**Sex**	
**Male**	24 (58.5%)
**Female**	17 (41.5%)
	**Median [Quartiles]**
**Age (years)**	40.0 [31.0, 46.0]
**Body height (cm)**	175.0 [164.0, 181.0]
**Body weight (kg)**	69.0 [60.0 85.0]
	**Total (%)**
**History of motion sickness**	13 (31.7%)
**Language**	
**German**	30 (73.2%)
**English**	11 (26.8%)
**Any AE or SAE**	1 (2.4%)
**Any protocol deviation**	1 (2.4%)

Supplementary Table S6

Analysis of the MSAQ summary score on the Intention to treat (ITT) and Per-Protocol (PP) set (A) and subscores on the PP set (B). We show the average change for each subscore with both techniques, and the difference between them. Confidence intervals are based on robust standard errors.
MSAQ: Motion sickness assessment questionnaire. DTRT: Dynamic trajectory radiotherapy, ncVMAT: Non-coplanar volumetric modulated arc therapy.

A)

	DTRT	ncVMAT	Difference
Intention to treat (N = 41)	1.88 (0.79 to 2.97)	1.62 (0.78 to 2.46)	0.26 (-0.24 to 0.75)
Per protocol (N = 40)	1.95 (0.83 to 3.08)	1.66 (0.80 to 2.51)	0.29 (-0.23 to 0.82)

B)

	DTRT (N = 40)	ncVMAT (N = 40)	Difference
Gastrointestinal subscore	0.22 (-0.02 to 0.46)	0.12 (-0.04 to 0.28)	0.10 (-0.04 to 0.25)
Central subscore	1.34 (0.57 to 2.12)	1.18 (0.59 to 1.78)	0.16 (-0.16 to 0.47)
Peripheral subscore	-0.10 (-0.16 to -0.05)	-0.06 (-0.13 to 0.00)	-0.04 (-0.11 to 0.03)
Sopite-related subscore	0.52 (0.17 to 0.88)	0.39 (0.12 to 0.66)	0.13 (-0.13 to 0.40)

Supplementary Figure S7

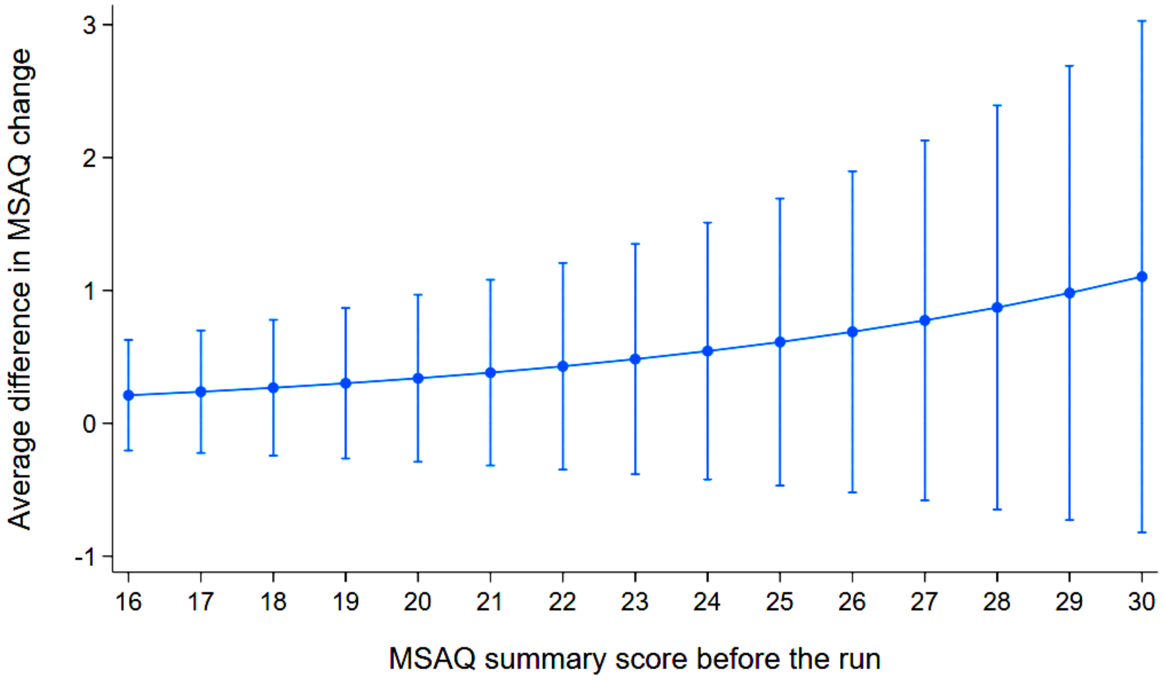

Supplementary Figure S10: Estimated difference with 95% confidence interval in MSAQ change between DTRT and ncVMAT techniques, given the baseline (before dry run) value of the MSAQ summary score. The summary score of 30 was the maximum observed baseline value.
MSAQ: Motion sickness assessment questionnaire.

Supplementary Figure S8

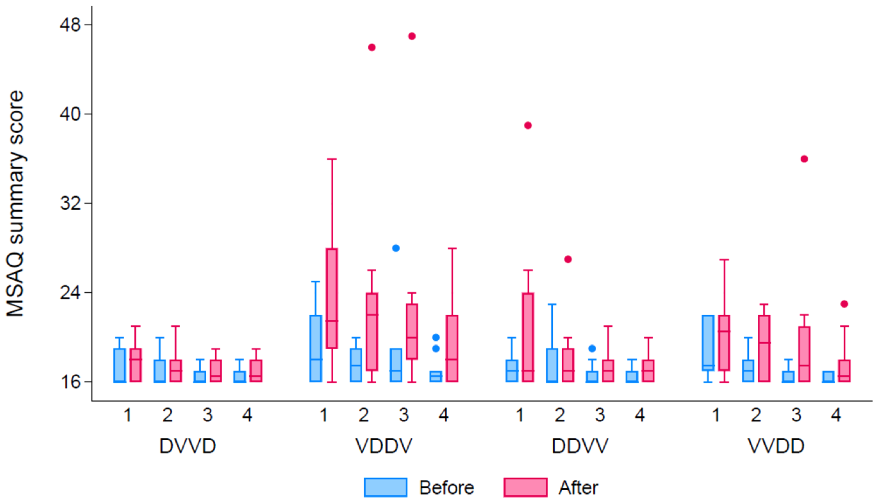

Supplementary Figure S11: Distribution of MSAQ summary score before and after each run, according to randomization groups and number of run (1-4).
MSAQ: Motion sickness assessment questionnaire. D: Dynamic trajectory radiotherapy (DTRT), V: Non-coplanar volumetric modulated arc therapy (ncVMAT).

Supplementary Figure S9

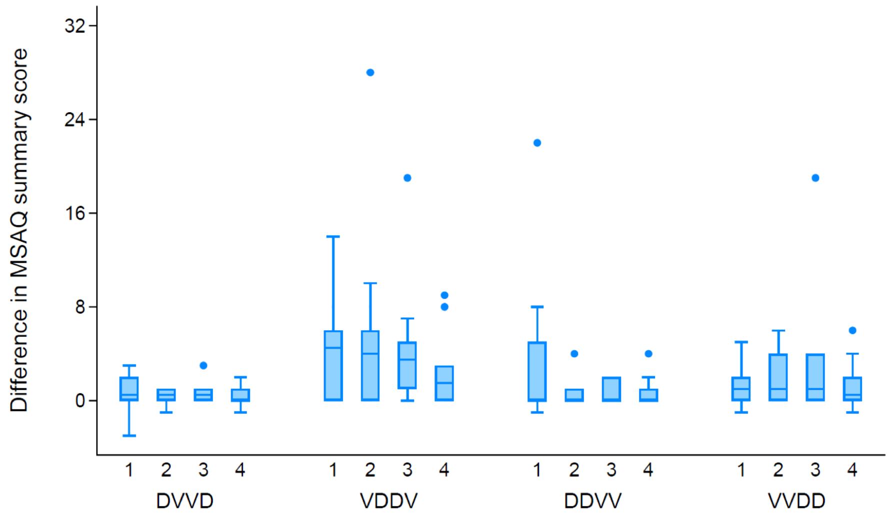

Supplementary Figure S12: Distribution of the difference in MSAQ summary score between after and before each run, according to randomization groups and number of run (1-4).
MSAQ: Motion sickness assessment questionnaire. D: Dynamic trajectory radiotherapy (DTRT), V: Non-coplanar volumetric modulated arc therapy (ncVMAT).

Supplementary Table S10

Descriptive statistics of the MSAQ summary score with mean and standard deviations, as well as median and quartiles of MSAQ summary score and MSAQ change for each run and each randomization group.
MSAQ: Motion sickness assessment questionnaire. D: Dynamic trajectory radiotherapy (DTRT), V: Non-coplanar volumetric modulated arc therapy (ncVMAT). sd: Standard deviation. lq: Lower quartile. uq: Upper quartile.

	DVVD (N = 10)	VDDV (N = 10)	DDVV (N = 11)	VVDD (N = 10)
MSAQ before run 1				
mean (sd)	17.3 (1.8)	19.1 (3.2)	17.1 (1.4)	18.6 (2.5)
median [lq, uq]	16.0 [16.0, 19.0]	18.0 [16.0, 22.0]	17.0 [16.0, 18.0]	17.5 [17.0, 22.0]
MSAQ after run 1				
mean (sd)	17.9 (1.9)	23.5 (6.4)	20.6 (7.0)	20.1 (3.7)
median [lq, uq]	18.0 [16.0, 19.0]	21.5 [19.0, 28.0]	17.0 [16.0, 24.0]	20.5 [17.0, 22.0]
MSAQ change at run 1				
mean (sd)	0.6 (1.6)	4.4 (4.5)	3.5 (6.8)	1.5 (2.1)
median [lq, uq]	0.5 [0.0, 2.0]	4.5 [0.0, 6.0]	0.0 [0.0, 5.0]	1.0 [0.0, 2.0]
MSAQ before run 2				
mean (sd)	16.9 (1.4)	17.5 (1.5)	17.4 (2.3)	17.2 (1.2)
median [lq, uq]	16.0 [16.0, 18.0]	17.5 [16.0, 19.0]	16.0 [16.0, 19.0]	17.0 [16.0, 18.0]
MSAQ after run 2				
mean (sd)	17.3 (1.6)	23.2 (8.7)	17.9 (3.3)	19.1 (2.7)
median [lq, uq]	17.0 [16.0, 18.0]	22.0 [17.0, 24.0]	17.0 [16.0, 19.0]	19.5 [16.0, 22.0]
MSAQ change at run 2				
mean (sd)	0.4 (0.7)	5.7 (8.5)	0.5 (1.2)	1.9 (2.3)
median [lq, uq]	0.5 [0.0, 1.0]	4.0 [0.0, 6.0]	0.0 [0.0, 1.0]	1.0 [0.0, 4.0]
MSAQ before run 3				
mean (sd)	16.4 (0.7)	18.1 (3.7)	16.5 (1.0)	16.5 (0.7)
median [lq, uq]	16.0 [16.0, 17.0]	17.0 [16.0, 19.0]	16.0 [16.0, 17.0]	16.0 [16.0, 17.0]
MSAQ after run 3				
mean (sd)	17.1 (1.3)	22.5 (9.0)	17.2 (1.5)	19.7 (6.1)
median [lq, uq]	16.5 [16.0, 18.0]	20.0 [18.0, 23.0]	17.0 [16.0, 18.0]	17.5 [16.0, 21.0]
MSAQ change at run 3				
mean (sd)	0.7 (0.9)	4.4 (5.6)	0.6 (0.9)	3.2 (5.8)
median [lq, uq]	0.5 [0.0, 1.0]	3.5 [1.0, 5.0]	0.0 [0.0, 2.0]	1.0 [0.0, 4.0]
MSAQ before run 4				
mean (sd)	16.5 (0.8)	17.0 (1.4)	16.4 (0.7)	16.4 (0.5)
median [lq, uq]	16.0 [16.0, 17.0]	16.5 [16.0, 17.0]	16.0 [16.0, 17.0]	16.0 [16.0, 17.0]
MSAQ after run 4				
mean (sd)	16.9 (1.1)	19.6 (4.3)	17.2 (1.3)	17.7 (2.5)
median [lq, uq]	16.5 [16.0, 18.0]	18.0 [16.0, 22.0]	17.0 [16.0, 18.0]	16.5 [16.0, 18.0]
MSAQ change at run 4				
mean (sd)	0.4 (0.8)	2.6 (3.3)	0.8 (1.3)	1.3 (2.2)
median [lq, uq]	0.0 [0.0, 1.0]	1.5 [0.0, 3.0]	0.0 [0.0, 1.0]	0.5 [0.0, 2.0]

Supplementary Table S11

Mean and standard deviations, as well as median and quartiles of MSAQ summary score and MSAQ change for each technique. For each measure (MSAQ before, after the run, and change), the intra-class correlation is shown, too. The latter is computed by considering a mixed model with the measure as outcome, and a random effect at participant level. Spearman's correlation between MSAQ summary score before and after the run is ρ = 0.69, with a 95% confidence interval from 0.6 to 0.76.

MSAQ: Motion sickness assessment questionnaire. D: Dynamic trajectory radiotherapy (DTRT), V: Non-coplanar volumetric modulated arc therapy (ncVMAT). ICC: Intra-class correlation. sd: Standard deviation. lq: Lower quartile. uq: Upper quartile.

	DTRT (N = 82)	ncVMAT (N = 82)	ICC (95% CI)
MSAQ before the run			0.34 (0.20 to 0.52)
mean (sd)	17.1 (1.9)	17.2 (1.9)	
median [lq, uq]	16.0 [16.0, 18.0]	16.0 [16.0, 18.0]	
MSAQ after the run			0.67 (0.54 to 0.78)
mean (sd)	19.5 (5.9)	18.8 (3.8)	
median [lq, uq]	17.5 [16.0, 21.0]	17.0 [16.0, 20.0]	
MSAQ change			0.59 (0.44 to 0.72)
mean (sd)	2.5 (5.1)	1.6 (2.6)	
median [lq, uq]	0.0 [0.0, 4.0]	1.0 [0.0, 2.0]	

Supplementary Table S12

Model coefficients, on both intention-to-treat (ITT, Full-Analysis Set) and per-protocol (PP) sets. As a reminder, the outcome is obtained by subtracting 15 to the MSAQ summary score after the runs ("modified MSAQ score" after the run).

MSAQ: Motion sickness assessment questionnaire. D: Dynamic trajectory radiotherapy (DTRT), V: Non-coplanar volumetric modulated arc therapy (ncVMAT).

	Full-Analysis Set	Per-Protocol Set
Modified MSAQ score at baseline	0.12 (0.05 to 0.19; p=0.001)	0.12 (0.05 to 0.19; p=0.001)
DTRT vs. ncVMAT	0.07 (-0.05 to 0.18; p=0.275)	0.07 (-0.05 to 0.20; p=0.237)
Run number (continuous)	-0.13 (-0.20 to -0.06; p<0.001)	-0.13 (-0.20 to -0.06; p<0.001)
Randomization result		
DVVD	0 (ref.)	0 (ref.)
VDDV	0.77 (0.18 to 1.36; p=0.010)	0.77 (0.18 to 1.36; p=0.010)
DDVV	0.14 (-0.34 to 0.63; p=0.559)	0.21 (-0.28 to 0.71; p=0.393)
VVDD	0.36 (-0.20 to 0.91; p=0.206)	0.36 (-0.20 to 0.91; p=0.206)
Intercept	*0.76 (0.37 to 1.15)*	*0.77 (0.38 to 1.16)*

References

[1] Jöhl A, Bogowicz M, Ehrbar S, Guckenberger M, Klöck S, Meboldt M, et al. Unconscious physiological response of healthy volunteers to dynamic respiration-synchronized couch motion. Radiat Oncol 2017;12:189. https://doi.org/10.1186/s13014-017-0925-6.

[2] McCullagh P, Nelder JA. Generalized Linear Models. 2nd Edition. New York: Routledge; 1989. https://doi.org/10.1201/9780203753736.

[3] StataCorp. Stata Statistical Software: Release 18 2023.

[4] R Core Team. R: A Language and Environment for Statistical Computing. Vienna, Austria: R Foundation for Statistical Computing; 2022.
